# Supplementary material for: Palau’s warmest reefs harbor thermally tolerant corals that thrive across different habitats
Source: Commun Biol. 2022 Dec 21;5:1394. doi: 10.1038/s42003-022-04315-7 (PMC9772186; doi:10.1038/s42003-022-04315-7)
Supplement: Supplementary file 2 — Supplementary Information [file 42003_2022_4315_MOESM2_ESM.pdf]

Supplementary Information

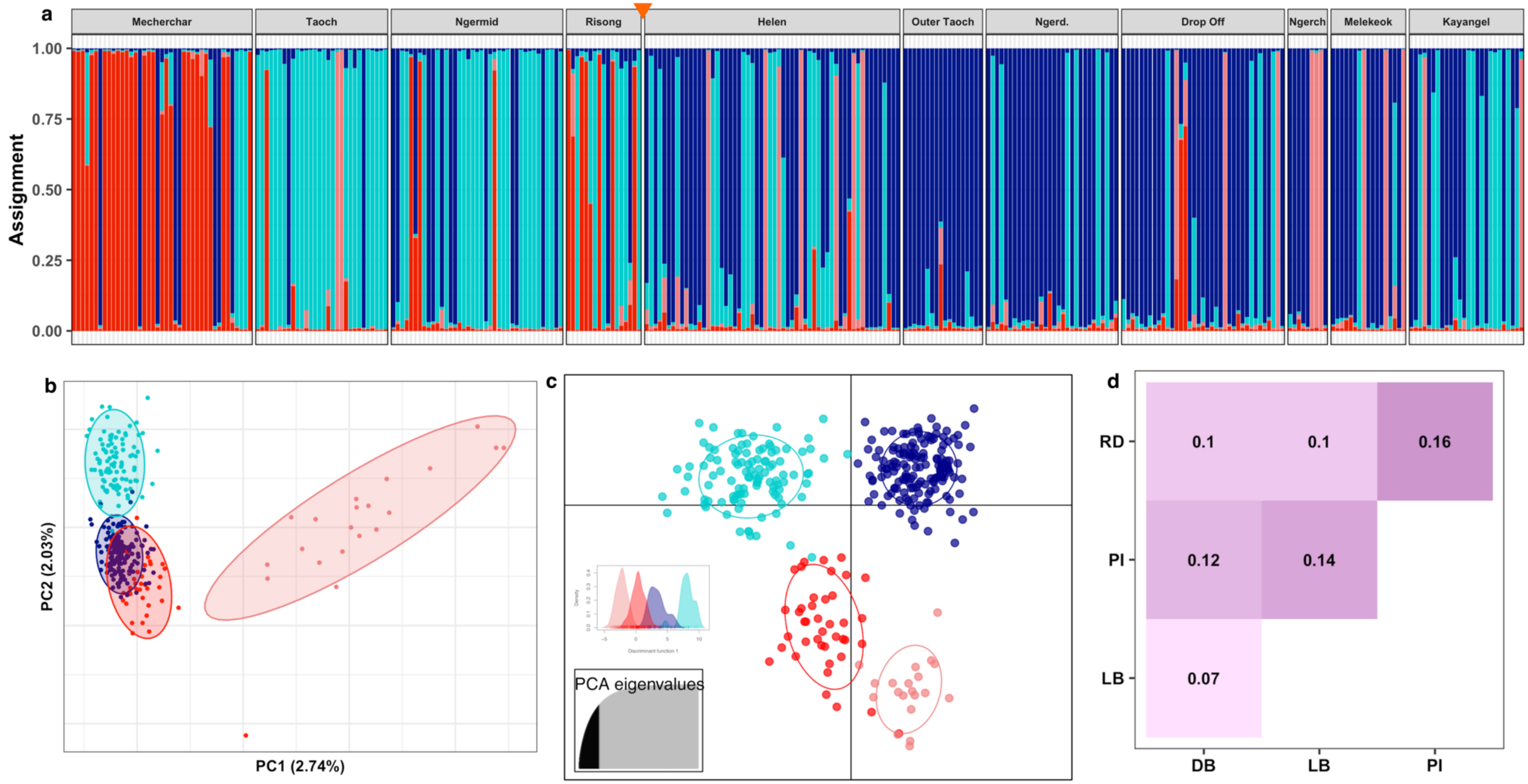

Figure S1. Microsatellite-based population structure. a) STRUCTURE for K=4. Rock Island sites are shown first (left of orange triangle), followed by outer reef sites (right of triangle). Rock Island sites are dominated by light blue (LB) and red lineages (RD) while the dark blue (DB) and pink lineages (PI) are more common on the outer reefs. **b)** Principal component analyses of microsatellite data. Points represent individual samples and are colored by their dominant lineage (>50% assigned lineage based on STRUCTURE results). **c)** Discriminant analysis of principal components (DAPC) recapitulates the four lineage clusters. Points are individual samples, colored by their lineage assignment. Top inset shows density across the first discriminant axis. Lower inset shows number of retained principal components for analysis (40) based on cross-validation optimization. **d)** Pairwise Nei's F_{ST} values between lineages. Color intensity increases with higher F_{ST}.

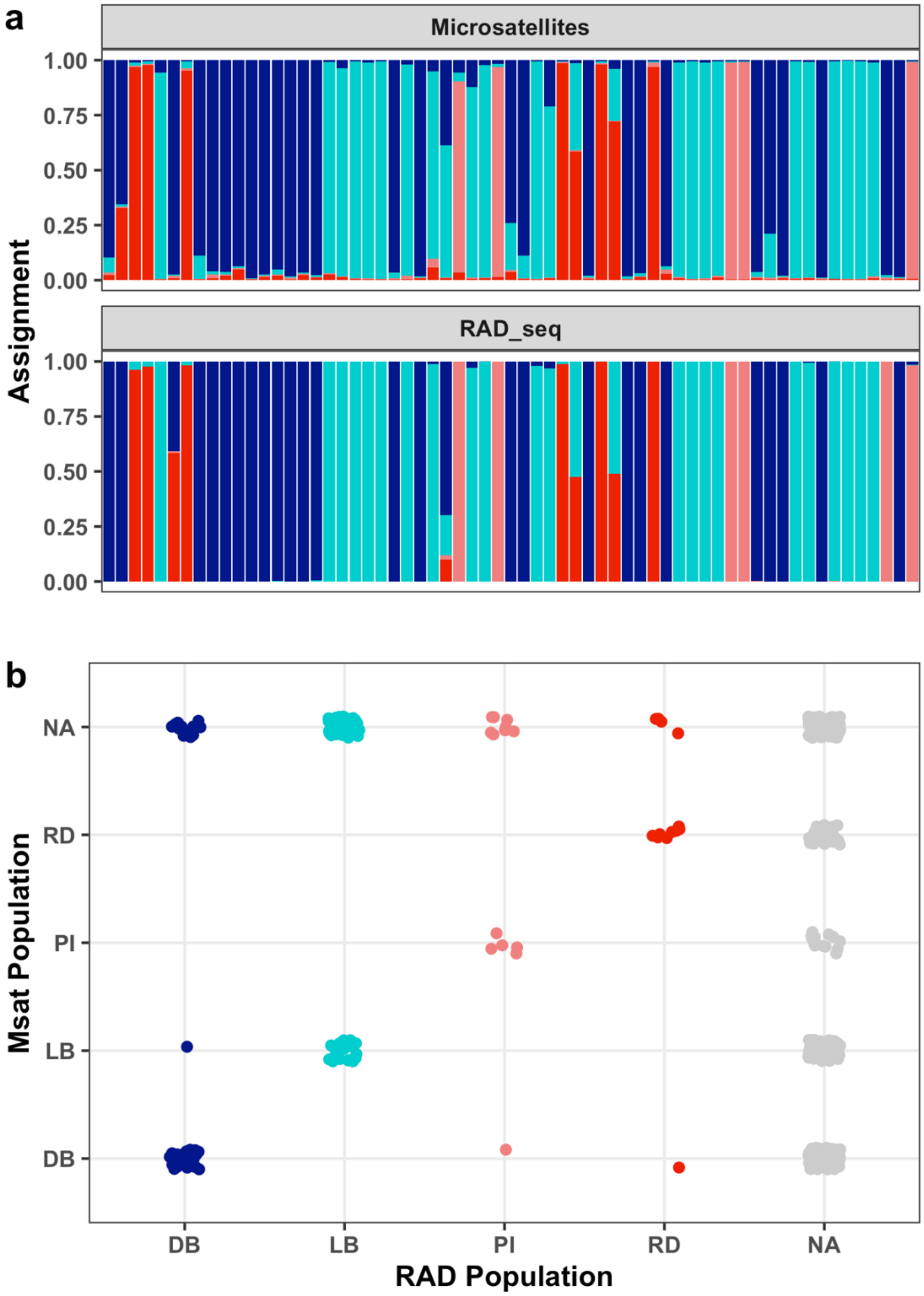

Figure S2. Comparison of STRUCTURE assignment results across samples using microsatellite and RAD-seq data. a) Ancestry assignments from STRUCTURE output for K=4 using an admixture model with correlated allele frequencies and default parameters. Bars are colored to represent the proportion of that sample's ancestry that is assigned to an ancestral population group. Individual samples are lined up across graphs. Three samples for which the predominant ancestral lineage switches between microsatellite and RAD-seq data are denoted by an asterisk. **b)** Sample lineages based on >50% assignment to an ancestral group. Lineage assignments agree between microsatellite-based and RAD-seq STRUCTURE results, except for the same 3 samples denoted by asterisks in panel A. Samples that were not analyzed by a particular method are denoted by NA.

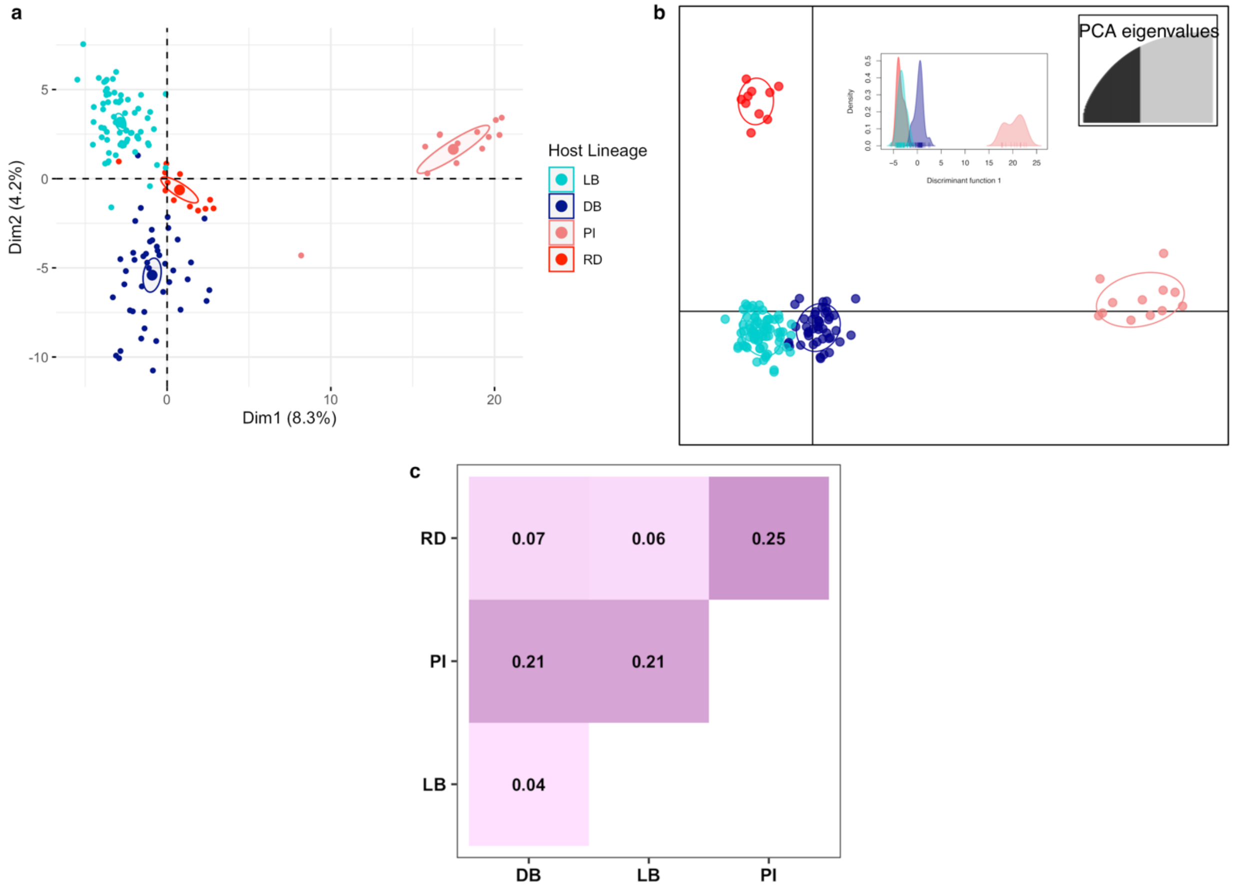

Figure S3. Population structure of symbionts based on RAD-seq SNPs. *N*=218 SNPs, from 147 samples. **a)** Principal component analyses. Points represent individual samples and are colored by the dominant lineage of the coral host (>50% assigned lineage based on STRUCTURE results as shown in Fig. 2). **b)** Discriminant analysis of principal components (DAPC) recapitulates the four lineage clusters seen in the coral hosts. Points are individual samples, colored by their coral host dominant lineage assignment. Top inset shows number of retained principal components for analysis (60) based on cross-validation optimization. **c)** Pairwise Nei's F_{ST} values between symbiont clusters based on coral host lineage groups. Background color intensity increases with higher F_{ST}. All F_{ST} values were significant based on 100 bootstrap calculations and alpha=0.05.

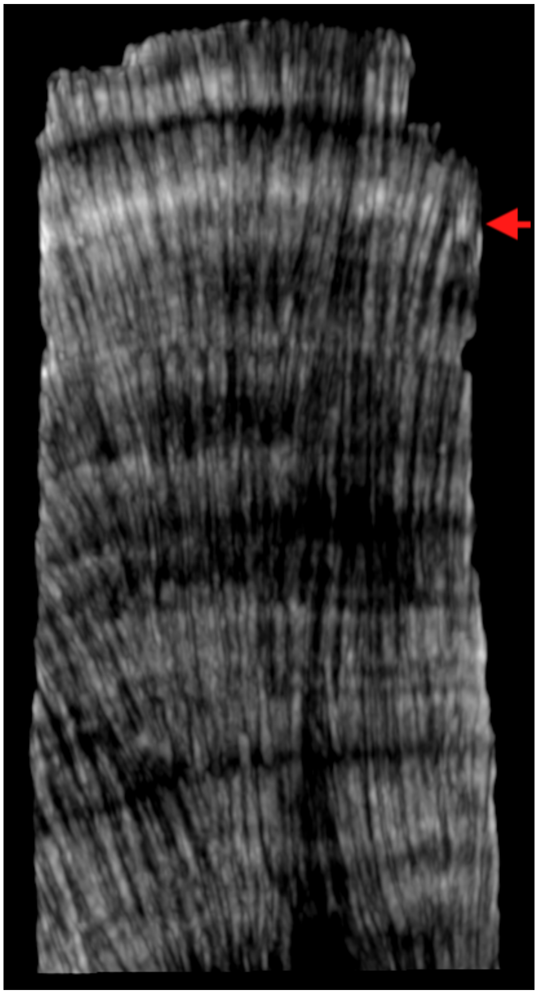

Figure S4. **Example of stress band in coral core.** CT image of a coral core collected in 2011, showing a 2010 stress band (red arrow). Higher density aragonite is brighter and whiter, while lower density bands are darker. Annual growth consists of high/low density couplets.

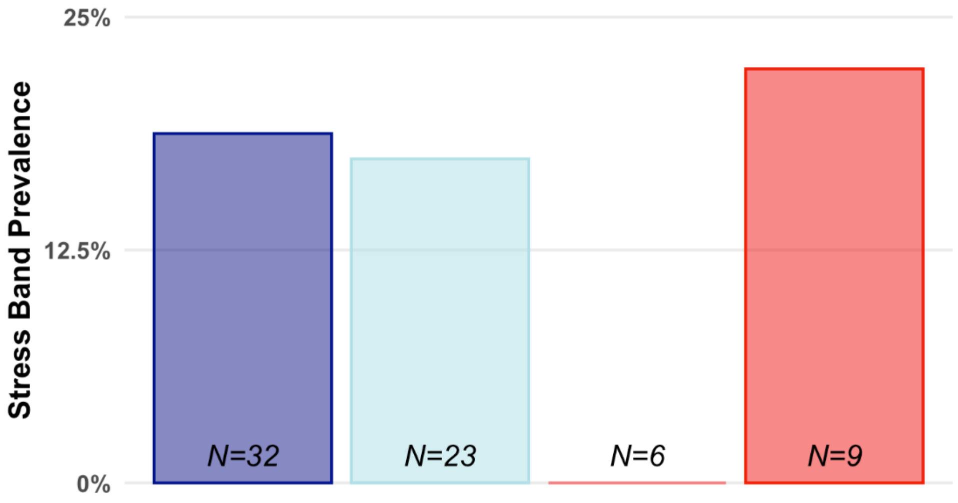

Figure S5. Stress band prevalence by lineage in 2010. N values denote the number of cores analyzed from each lineage. Note that sample size is slightly larger here than in Fig. 4D, as not all the cores collected extended back to 1998.

Site	Sample	Other samples with same bands (from various sites)	Hit
Risong	Palau034	Palau234, 266, 245, 242, 041, 112, 113, 053, 294, 314	*Symbiodinium* sp. C15 isolate POR3B 5.8S ribosomal RNA gene, partial sequence; internal transcribed spacer 2, complete sequence; and 28S ribosomal RNA gene, partial sequence
Ngermid	Palau113	Palau266, 034, 112, 041	
Helen	Palau171	Palau213, 200, 176, 053, 054, 061, 062, C15	
Mecherchar	Palau264	Palau 270,354, 268	
	Palau270	Palau264, 293, 306, 308, 354, 268	
Mecherchar	Palau266	Palau034,112, 041	
Isolate 5<br>Isolate 7	C15 Isolate<br>C15 Isolate	All	
Mecherchar	Palau270	Palau 306,308,346,354	*Symbiodinium* sp. C clone KB184RR_3 5.8S ribosomal RNA gene, partial sequence; internal transcribed spacer 2, complete sequence; and 28S ribosomal RNA
		Palau 264,306,308,348	*Symbiodinium* sp. clade C isolate Colony-7D1 5.8S ribosomal RNA gene, partial sequence; internal transcribed spacer 2, complete sequence; and 28S ribosomal RNA gene, partial sequence

Supplementary Table 1. *Symbiodinium* DGGE genotyping results. BLAST hits of ITS2 sequences amplified from bands extracted from DGGE of the ITS2 region. All samples analyzed shared bands with the C15 culture isolates. Additional bands were likely heterodimers but were extracted and sequenced to verify genus identity. All results correspond to *Cladocopium* symbionts, primarily ITS2-type C15.

Supplementary Data 1. Excel spreadsheet showing pairwise F_{ST} values between sites for each lineage. The first tab shows F_{ST} based on RAD SNPs, the second based on microsatellite data. Comparisons for which the number of samples is <5 for at least one site are highlighted as these values more unreliable due to small sample size.

Supplementary Data 2. Comma-separated file, showing sample metadata information. Includes sample name, site of collection, the region (outer reef (OR) or Rock Island (RI)), latitude and longitude in decimal degrees, collection date, and colony depth (rounded to the nearest tenth of a meter).

Supplementary Data 3. Tab-separated file, showing coral core growth data by year. Includes sample name, site of collection, the core ID number, the year of growth, and average density, extension, and calcification values for each year analyzed. The same file is also available in the github repository, where it is called 'core_data.txt'

Supplementary Data 4. Tab-separated file, showing sampling metadata and stress band data. Includes sample name, cohort, site of collection, the region (outer reef (OR) or Rock Island (RI)), latitude and longitude in decimal degrees, collection date, and colony depth (rounded to the nearest tenth of a meter, colony diameter (in meters), and the presence (coded as 1) or absence (coded as 0) of stress bands in 1998 and 2010 (colonies without SB data are coded as NA). The same file is also available in the github repository, where it is called 'Strata_all_samples.txt'
