# Supplementary material for: Palau’s warmest reefs harbor thermally tolerant corals that thrive across different habitats
Source: Commun Biol. 2022 Dec 21;5:1394. doi: 10.1038/s42003-022-04315-7 (PMC9772186; doi:10.1038/s42003-022-04315-7)
Supplement: Supplementary file 3 — Description of Additional Supplementary Files [file 42003_2022_4315_MOESM3_ESM.pdf]

Description of Additional Supplementary Files

Supplementary Data 1: Excel spreadsheet showing pairwise FST values between sites for each lineage

Supplementary Data 2. Comma-separated file, showing sample metadata information

Supplementary Data 3. Tab-separated file, showing coral core growth data by year.

Supplementary Data 4. Tab-separated file, showing sampling metadata and stress band data
