# Supplementary material for: Palau’s warmest reefs harbor thermally tolerant corals that thrive across different habitats
Source: Commun Biol. 2022 Dec 21;5:1394. doi: 10.1038/s42003-022-04315-7 (PMC9772186; doi:10.1038/s42003-022-04315-7)
Supplement: Supplementary file 8 — Reporting Summary [file 42003_2022_4315_MOESM8_ESM.pdf]

nature portfolio

Corresponding author(s): Hanny E Rivera, Anne L Cohen

Last updated by author(s): Nov 13, 2022

Reporting Summary

Nature Portfolio wishes to improve the reproducibility of the work that we publish. This form provides structure for consistency and transparency in reporting. For further information on Nature Portfolio policies, see our Editorial Policies and the Editorial Policy Checklist.

Statistics

For all statistical analyses, confirm that the following items are present in the figure legend, table legend, main text, or Methods section.

n/a	Confirmed	
☐	☒	The exact sample size (*n*) for each experimental group/condition, given as a discrete number and unit of measurement
☐	☒	A statement on whether measurements were taken from distinct samples or whether the same sample was measured repeatedly
☐	☒	The statistical test(s) used AND whether they are one- or two-sided<br>*Only common tests should be described solely by name; describe more complex techniques in the Methods section.*
☒	☐	A description of all covariates tested
☐	☒	A description of any assumptions or corrections, such as tests of normality and adjustment for multiple comparisons
☐	☒	A full description of the statistical parameters including central tendency (e.g. means) or other basic estimates (e.g. regression coefficient) AND variation (e.g. standard deviation) or associated estimates of uncertainty (e.g. confidence intervals)
☐	☒	For null hypothesis testing, the test statistic (e.g. *F*, *t*, *r*) with confidence intervals, effect sizes, degrees of freedom and *P* value noted<br>*Give P values as exact values whenever suitable.*
☐	☒	For Bayesian analysis, information on the choice of priors and Markov chain Monte Carlo settings
☒	☐	For hierarchical and complex designs, identification of the appropriate level for tests and full reporting of outcomes
☒	☐	Estimates of effect sizes (e.g. Cohen's *d*, Pearson's *r*), indicating how they were calculated

Our web collection on statistics for biologists contains articles on many of the points above.

Software and code

Policy information about availability of computer code

Data collection	Versions of all software used is specified in the methods section. Listed here for reference:<br>GENEMAPPER™ v3.0 (Applied Biosystems), Stacks v.1.46, dDocent v.2.3.7 wrapper [CD-HIT v.4.6.8, BWA v.0.7.17, FreeBayes v.1.1.0] , vcftools v.0.1.15, and vcffilter (no version number available, single release), STRUCTURE v.2.3.4, R v.4.1.1. Version of individual R packages are included as comments at the end of scripts. Because R utilizes so many different packages listing these all here would be counterproductive. These are listed on the github repository however.
Data analysis	All scripts used to analyses the data are available at https://github.com/hrivera28/Palau_porites along with necessary data input files, and figure outputs. The metadata file (Supplementary_Data_1.csv) is also included in this repository. Raw sequencing data are available in the NCBI SRA, under accession # PRJNA801929

For manuscripts utilizing custom algorithms or software that are central to the research but not yet described in published literature, software must be made available to editors and reviewers. We strongly encourage code deposition in a community repository (e.g. GitHub). See the Nature Portfolio guidelines for submitting code & software for further information.

Data

Policy information about availability of data

All manuscripts must include a data availability statement. This statement should provide the following information, where applicable:

- Accession codes, unique identifiers, or web links for publicly available datasets
- A description of any restrictions on data availability
- For clinical datasets or third party data, please ensure that the statement adheres to our policy

All data associated with this manuscript are available in the supplementary information or appropriate databases: RAD-sequencing data are available on NCBI's SRA

March 2021

under accession number PRJXXXX. Scripts and other input data are available in the github repository of the first author at https://github.com/hrivera28/Palau_porites. The only exception (due to large memory requirements) is for raw coral core CAT scan files, which are available upon request to ALC. Coral sampled were imported to the U.S. under CITES permit numbers: PW14-163, PW15-022, PW18-121.

Field-specific reporting

Please select the one below that is the best fit for your research. If you are not sure, read the appropriate sections before making your selection.

☐ Life sciences ☐ Behavioural & social sciences ☒ Ecological, evolutionary & environmental sciences

For a reference copy of the document with all sections, see nature.com/documents/nr-reporting-summary-flat.pdf

Ecological, evolutionary & environmental sciences study design

All studies must disclose on these points even when the disclosure is negative.

Study description	Porites lobata colonies were sampled across 11 reefs across Palau. DNA was extracted from tissue samples for microsatellite genotyping and single nucleotide polymorphism sequencing (RAD-seq). In addition, coral cores for a subset of samples were collected to examine historical growth and thermal stress responses.
Research sample	Between 2011-18, we collected tissue from 543 Porites cf. lobata coral colonies using a hammer and chisel while on SCUBA. Coral cores were taken for 80 samples using an underwater pneumatic drill equipped with a diamond-tipped drill bit powered by compressed air from a SCUBA tank. Cores ranged from 10 to 204 cm long. Location information for each sample (lat/long, depth, date, site) are available in tableS2.csv.
Sampling strategy	Colonies were sampled as they were encountered on the reef for tissues. For coring, larger colonies, with a well rounded morphology and live tissue at the apex of the mound were preferred to ensure coring would be possible along the main growth axis. A target of 30 samples per site was the goal during sampling as this is general rule of thumb in population genetics analyses of diploid organisms.
Data collection	Trained scientific divers collected coral tissue samples and cores. Of the authors, H.E. Rivera and K. M-K. were involved in sample collection. Other individuals that assisted in sample collection are noted in the acknowledgments.
Timing and spatial scale	Samples were collected between 2011 and 2018 over the course of several field expeditions to Palau. Exact collection dates for each sample are specified in tableS2.csv.
Data exclusions	Samples were excluded from analyses if sufficient quality DNA could not be extracted from the sample or if the microsatellite or SNP genotyping data for that sample failed to pass quality and filtering steps detailed in the methods section.
Reproducibility	The results from the data inputs provided as part of this publication are reproducible and include all necessary scripts and files to replicate. Reproduction of the study itself would require sampling of corals colonies across the same sites and analyses of a similar fashion to those used here. The sampling information and data analyses pipelines we provide would be sufficient for reproduction. However, as the individuals sampled would not be exact same results and conclusions could vary.
Randomization	This was not applicable to this study. Samples were collected from coral colonies in their native sites and were not subjected to any experimental treatments. The contrasts used in this study are differences between natural sites. For sequencing purposes samples were individually barcoded and pooled across sequencing lanes to prevent lane/batch effects on sequencing data.
Blinding	Blinding is not particularly relevant to this study. The more subjective analyses presented are scoring of microsatellite alleles were are done automatically but verified and corrected by a person. The data in this case had only sample names of increasing number order without any designation from which site the sample was from, which could be a contrast variable for population genetics.

Did the study involve field work? ☒ Yes ☐ No

Field work, collection and transport

Field conditions	Temperature data for most sites are presented as part of this manuscript.
Location	Samples were collected across the country of Palau.
Access & import/export	Corals are protected under CITES. Marine research and collection permits were obtained from the government of Palau. Fees for operating within the Rock Islands area were also paid to the local government. CITES permit numbers: PW14-163, PW15-022, PW18-121.
Disturbance	Coreholes were plugged with a concrete chip (pre-made to fit in the exact core diameter) and affixed with underwater epoxy. This methods allow the surrounding coral tissue to regrown over the area and continue colony growth.

Reporting for specific materials, systems and methods

We require information from authors about some types of materials, experimental systems and methods used in many studies. Here, indicate whether each material, system or method listed is relevant to your study. If you are not sure if a list item applies to your research, read the appropriate section before selecting a response.

Materials & experimental systems

n/a	Involved in the study
☒	☐ Antibodies
☒	☐ Eukaryotic cell lines
☒	☐ Palaeontology and archaeology
☒	☐ Animals and other organisms
☒	☐ Human research participants
☒	☐ Clinical data
☒	☐ Dual use research of concern

Methods

n/a	Involved in the study
☒	☐ ChIP-seq
☒	☐ Flow cytometry
☒	☐ MRI-based neuroimaging
